# Thermophysiological Comfort Assessment of a Skirt Made from Bio-Based Material Derived from Pineapple Leaves

**DOI:** 10.3390/ma18143249

**Published:** 2025-07-10

**Authors:** Marija Pešić, Ineta Nemeša, Danka Đurđić, Dijamanta Salihi

**Affiliations:** Technical Faculty “Mihajlo Pupin”, University of Novi Sad, 23000 Zrenjanin, Serbia; inetavil@gmail.com (I.N.); dankadjurdjicsd@gmail.com (D.Đ.); dijamanta.812@gmail.com (D.S.)

**Keywords:** bio-based material, thermophysiological comfort, subjective comfort, thermal insulation, water vapor resistance

## Abstract

The purpose of this paper is to evaluate the thermophysiological comfort of pineapple bio-based nonwoven material as a sustainable alternative to natural leather and synthetic polymer-coated materials by analyzing both the objective parameters of the material and subjective user feedback by wearing a skirt made from the same material. Considering the increasing demand for sustainable materials alternatives, the study aims to determine whether this material can offer acceptable comfort during wear. The research included two commercially available pineapple, bio-based, nonwoven materials that differed in their finishing. Sample S1 contained 5% Bio-PU and 5% conventional PU, and sample S2 contained 10% conventional PU. Objective parameters such as thermal resistance (R_ct_), water vapor resistance (Ret) and air permeability were measured. For the subjective evaluation, ten female subjects wore the pineapple bio-based material skirts under controlled environmental conditions. Sample S1 showed lower R_ct_ values and slightly lower Ret combined with higher air permeability, which correlated with better subjective comfort evaluation. Although both samples showed high R_et_ values (S1 = 60.57 Pa^2^/W; S2 = 84.80 m^2^K/W) indicating limited vapor transfer, sample S1 was perceived as more comfortable, which was effected by better air permeability (S1 = 11.3 mm/s; S2 = 2.65 mm/s). Overall, S1 is more suitable for indoor use and for a shorter wear duration, while S2 may be better for cooler outdoor environments.

## 1. Introduction

In recent decades, concerns regarding sustainability have become a key factor in industrial production, and the fashion and textile industries are no exception. The pressure to reduce the negative impact on the environment, together with the problems of climate change, resource depletion and pollution, has led to the need for a wider use of natural and biodegradable materials, as well as a reduction in dependence on fossil fuels [1]. One of the key steps towards sustainable textile production is the replacement of petroleum-based synthetic materials with natural-origin materials, which are more environmentally beneficial. “Green” principles are becoming the basic business strategies of brands that are increasingly turning to sustainable alternatives and new materials [2,3]. Consumers increasingly value sustainability as a key factor when making purchasing decisions [3,4].

Leather is one of the oldest materials, with a long history of use. It is widely used for clothing, footwear, handbags, furniture, tools and sports equipment manufacturing [5]. The production of both natural leather and synthetic polymer-coated materials, which are commonly used as leather alternatives, has a negative impact on the environment and human health. Natural leather also involves the unethical treatment of animals. Increased consumer awareness of environmental and ethical aspects, as well as potential health risks, has led to a greater interest in sustainable alternative materials such as bio-based materials. They can be produced from renewable and biodegradable sources, such as pineapple, apple, cactus, mango and others. These bio-based leather alternatives offer a more environmentally friendly solution and enable more ethical and humane production [6,7,8,9].

In the past, sustainable clothing was often associated with compromises in comfort and aesthetics, but thanks to technological advances, today there is no longer a choice between environmental responsibility and comfort. Generation Z and millennials (born between the 1980s and early 2000s) are bringing significant changes to the fashion industry by choosing brands that are ethical and environmentally conscious and that offer quality clothing that lasts [3,4,10].

There is currently a limited amount of research dealing with the thermophysiological comfort of clothing made from pineapple bio-based material. Most existing studies focus on the manufacturing process and physical–mechanical characteristics of this material, such as strength, durability and wear resistance [11,12,13,14,15,16].

The feeling of clothing comfort is a subjective state of mind that creates a balanced process of heat exchange between the human body, clothing and the environment [17].

Thermophysiological comfort of clothing refers to its ability to manage heat and moisture transfer in a way that helps maintain the body’s thermal balance, both at rest and during physical activities of varying intensity [17]. According to the ISO 7730 standard, thermal comfort is defined as “a state of mind that expresses satisfaction with the warmth of the environment” [18,19,20,21].

Heat in the human body is generated through isothermal biochemical processes—metabolism. The body’s total heat is composed of the basic amount of heat, basal heat, which is produced independently of human activity, and the amount of heat that depends on a person’s physical activity, working heat. It can be said that heat production depends on the type of activity, age and gender, as well as the type and amount of clothing the subject is wearing [22].

The aim of this paper is to examine the possibility of expanding the use of pineapple bio-based material as a leather alternative in clothing, analyzing its thermophysiological characteristics and the impact on clothing comfort. A special focus will be put on the comparison of subjective and objective thermophysiological comfort, in order to determine whether this material can meet the requirements of functional and modern clothing, while maintaining sustainability and environmental friendliness.

## 2. Materials and Methods

### 2.1. Materials

Pinatex is a nonwoven textile material made from pineapple leaf fibers (PALF), and was developed by the company Ananas Anam Ltd. (www.ananas-anam.com, accessed on 20 April 2025) in 2015. The material is produced from agricultural waste, which is generated after the pineapple harvest. The fibers are combined with corn-based polylactic acid (PLA) and treated mechanically, forming Pinafelt—the non-woven web that forms the basis of Pinatex material [7,8,9,11,23].

Although pineapple bio-based material is mainly used to make footwear and fashion accessories, more and more, well-known fashion designers, such as Evelyn Fink [24], Laura Strambi [25] and Liselore Frowijn [23], have found its use in outerwear, including skirts, dresses and jackets [7]. The two variants that are chosen for this study are among the most commonly used Pinatex types in the production of apparel.

In this study, two commercially available variants of Pinatex material were used to analyze the subjective and objective comfort ratings:

- Pinatex Original (S1), a non-woven textile material of 72% PALF 18% PLA, 5% BIO PU, 5% PU;
- Pinatex Metallic (S2), a non-woven textile material of 72% PALF, 18% PLA, 10% PU.

**Figure 1:**
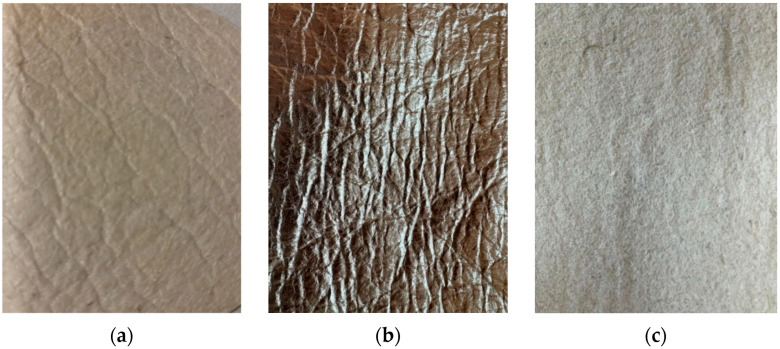
Pinatex—pineapple bio-based material: (**a**) sample S1 face side, (**b**) sample S2 face side, (**c**) back side (figure created by the authors).

The basic characteristics of this materials are presented in Table 1, which includes values for surface mass, thickness, air permeability and thermal and evaporative resistance. Figure 1 provides a visual representation of the tested materials.

### 2.2. Tested Garment

Pinatex, due to its thickness and structural strength, is most often used to make pieces like jackets and skirts or as details/smaller parts on dresses and blouses, which can be confirmed through examples in contemporary fashion design. The skirt, in this case, was chosen as the optimal solution because it can be worn in different conditions—both indoors and outdoors—thus enabling wider testing of the environmental impact on the thermophysiological comfort of the material.

**Figure 2:**
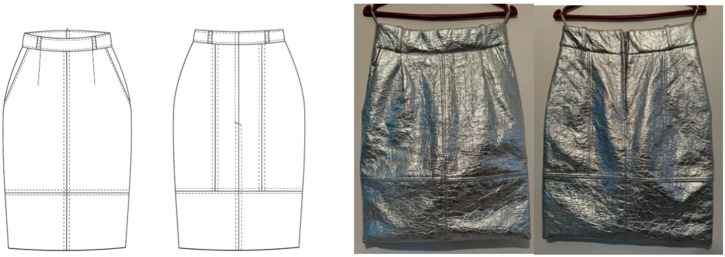
Skirt model made from pineapple bio-based material for the subjective comfort evaluation (figure created by the authors).

At the time of the study, the specified skirt model (see Figure 2) made from pineapplebio-based material, combined with a cotton blouse, was worn by the ten test subjects fiting size 38.

## 3. Methods

### 3.1. Objective Methods of Determining the Thermophysiological Comfort of Clothing

In order to analyze the thermophysiological comfort of pineapple bio-based material, objective testing methods were used that allow quantitative assessment of the material’s ability to regulate heat and moisture between the body and the environment. Pineapple bio-based material samples S1 and S2 were tested under standard conditions, where the air humidity was 65 ± 4%, and the temperature was 20 ± 2 °C, according to the ISO 139:2005 standard [28].

Air permeability is defined as the rate of air flow through a defined surface area of a material at a given pressure difference. The test was conducted according to ISO 9237:1995 [29] using the TexTest FX3300 device (Textest AG, Schwerzenbach, Switzerland), at a pressure of 100 Pa, on an area of 20 cm^2^.

The thermal resistance (R_ct_) is the thermal insulation of the material and is inversely proportional to the thermal conductivity, as shown by Equation (1) [21,30].

(1) $$R_{ct}=\frac{h}{\lambda }\left(m^{2}K/W\right)$$

In dry materials or in materials containing very little water, R_ct_ depends on the thickness of the material (h) and the conductivity of the fibers (λ).

The thermal conductivity coefficient is calculated according to Equation (2) [21,30]:

(2) $$\lambda =\frac{ɸ\cdot h}{A_{BT}(T_{BT}-T_{a})}\;or\;\lambda =\frac{ɸ\cdot h}{A_{BT}\cdot ∆T}$$

where λ—thermal conductivity coefficient $\left[W/mK\right]$, $A_{BT}$—plate surface $\left[0.0025\;m^{2}\right]$, *h*—material thickness $\left[m\right]$, $T_{BT}$—plate temperature $\left[K\right]$, $T_{a}$—ambient temperature $\left[K\right]$, ɸ—heat flow $\left[W\right]$.

Water vapor resistance (Ret) represents the resistance of textile material to the flow of water vapor, i.e., a measure of how difficult the material is to evaporate sweat from the surface of the skin.

Water vapor resistance Ret was determined according to the following Equation (3) [21,30]:

(3) $$R_{et}=\frac{\left(p_{s}-p_{a}\right)\cdot A}{H_{et}}$$

where is: $R_{et}-water\;vapor\;resistance\;\left[\;\mathrm{Pa}m^{2}W^{-1}\right],\;H_{et}$—evaporated heat flow $[W],\;p_{s}$—partial pressure on the surface of plate [Pa], $p_{a}$—partial air pressure in wind tunnel $\left[Pa\right],\;A$—plate surface $\left[m^{2}\right]$.

In this research, thermal resistance and water vapor resistance were measured using a heat plate that replaced a human body. The air temperature during the experiment was 20 °C; the plate temperature was 35 °C. The measurements were made according to the ISO 11092:2014 standard [31]. Test samples with dimensions of 300 × 300 mm were used for testing the thermal insulation.

### 3.2. Subjective Methods of Evaluating Thermophysiological Comfort of Clothing

#### 3.2.1. Test Subjects in the Evaluation of Subjective Thermophysiological Comfort

For the testing of subjective thermophysiological comfort, ten female subjects of average build and height ranging from 159 to 174 cm were selected (Table 2). Although a larger number of test subjects would provide a broader generalization of the results, this number of test subjects was selected in order to identify relevant trends and gain insight into the comfort potential of the analyzed pineapple materials. The primary goal of the research was to uncover indicative patterns, rather than draw conclusions at the population level.

All of the selected subjects for testing were in good general health, without allergies to textile materials of different origins and chemicals used in the processing of Pinatex materials. One hour before the examination, the subjects did not eat and took care of sufficient fluid intake.

Based on data of height and body mass, the body surface area and ideal mass of each tested subject was calculated.

#### 3.2.2. Experimental Conditions for the Assessment of Subjective Thermophysiological Comfort

The subjective feeling of thermal comfort was evaluated based on a questionnaire and appropriate scales according to the ISO 10551 standard [32,33,34]. The room temperature of 20–23 °C and air humidity of 60–65% were chosen, the same as when measuring the objective thermophysiological comfort of the tested skirts.

The testing protocol under controlled conditions was in the fallowing order: 15 min of acclimatization and rest in an air-conditioned room, then 20 min of walking in the room and 20 min of sitting and rest. After each phase of testing, the subjects filled out a questionnaire about their subjective feeling of thermal comfort.

#### 3.2.3. Questionnaire for Assessing Subjective Thermophysiological Comfort

In order to properly assess the subjective feeling of heat inside the room of the subjects who participated in the experiment, before the start of the test, during and after the test, a questionnaire with 5 questions was prepared. The goal of the questionnaire was to determine the influence of the thermal environment and clothing on the overall thermal condition of the respondents during their stay and movement inside the room. The offered answers to the questions are given in the form of a scale where 0 marks the starting point, 1, 2, 3 and 4 represent the degrees of intensity of discomfort in terms of heat, and −1, −2, −3 and −4 in terms of cold. A score of 0 is taken as complete comfort and corresponds to 100% satisfaction, while a score of 4 indicates 0% satisfaction or a complete lack of comfort felt by the respondent.

The questions in the questionnaire were as follows:

- A: How would you rate your current sensation of warmth?
- B: How would you describe your current level of thermal comfort?
- C: What change in the thermal environment would you prefer?
- D: In your opinion, how acceptable is the current thermal environment?
- E: How well are you tolerating the current thermal conditions?

## 4. Results and Discussion

The following parameters were calculated: thermal resistance (R_ct_), water vapor resistance (R_et_), thermal conductivity coefficient and air permeability.

Both tested samples (S1, S2) have the same content of PALF fibers and PLA, but they have different type of finishing. The sample S1 was treated with a top coat containing 5% bio-polyurethane (bio-PU) and 5% conventional PU, while sample S2 has 10% conventional PU coating. This difference affects both the objective and subjective measurement results. In Table 3, the thermophysiological properties of analyzed samples are presented.

According to the results of the standard deviation (SD) in Table 3, the biggest variability is in air permeability, due to the uneven porosity structure of the material. The thermal and vapor resistance parameters showed a moderate degree of deviation, which indicates relatively more uniform measurements of these properties.

Sample S1, despite its greater thickness, shows a lower surface mass compared to sample S2. This can be explained by the different density and structure of the finishing treatments: the bio-PU in the sample S1 can contribute to a lower density and higher porosity, which results in a lower total mass. On the other hand, sample S2, with 10% conventional PU, may have a more compact and dense structure, which contributes to a higher surface mass despite the lower thickness. This greatly affects the air permeability (AR) and porosity of the material. Air permeability for sample S1 is 11.3 mm/s, and for sample S2 it is 2.65 mm/s, and it directly affects the thermal insulation (R_ct_) and the subjective feeling of thermal comfort, especially in conditions of physical activity. Higher surface mass is associated with lower air permeability.

**Figure 3:**
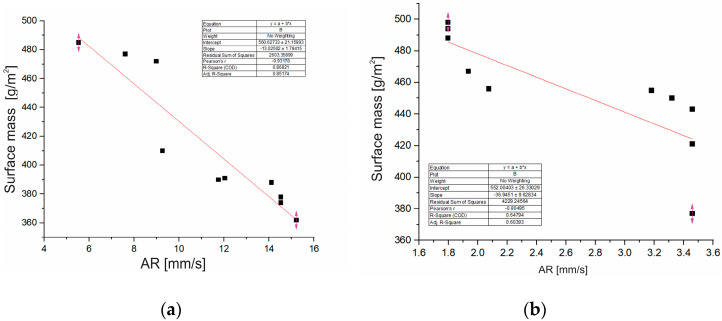
Dependence of surface mass on air permeability (AR) for (**a**) sample S1, (**b**) sample S2 (graphs created by the authors based on experimental data).

Linear regression analysis (Figure 3) revealed a statistically significant negative relationship between air permeability and surface mass for sample S1, r = –0.93, R^2^ = 0.85, and for sample S2, r = –0.80, R^2^ = 0.65. The obtained regression model suggests that an increase in surface mass was associated with a decrease in air permeability. Furthermore, results from a two-sample *t*-test confirmed that the difference in air permeability between sample S1 and sample S2 is statistically significant (t = 8.01, *p* < 0.0001). This indicates that the presence of a combination of bio-based PU and conventional PU in S1 contributes to a more breathable structure compared to S2.

By comparing the values of thermal resistance (R_ct_) and water vapor resistance (Ret), it was determined that sample S1 (R_ct_ = 0.0106 m^2^K/W; R_et_ = 60.57 m^2^Pa/W) shows more desirable thermal properties compared to sample S2 (R_ct_ = 0.0257 m^2^K/W; Ret = 84.80 m^2^Pa/W). The R_ct_ values are lower for both samples, which indicates good heat dissipation. Lower values of Ret in sample S1 allow a slightly easier evaporation of water vapor. High values of the water vapor resistance (R_et_) indicate a weak ability to evaporate sweat. Although the Ret values of both samples are high and exceed the recommended limits for optimal comfort, sample S1 shows good air permeability and a lower insulating effect, which allows a certain degree of ventilation and removal of heat and sweat, even in conditions when Ret is very high, which makes it more suitable for wearing in warmer (room) conditions and physically active conditions, unlike sample S2.

**Figure 4:**
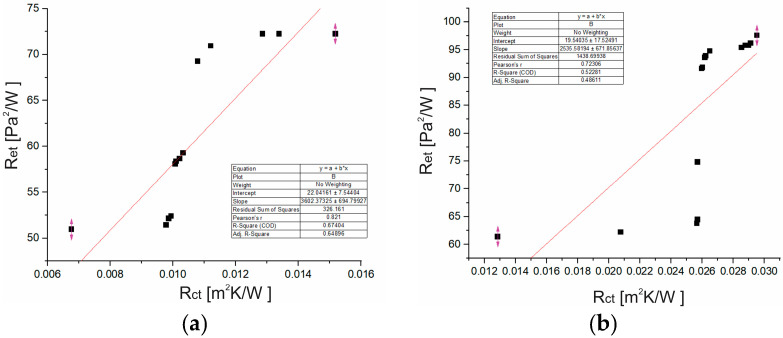
Dependence of R_et_ on R_ct_ for (**a**) sample S1, (**b**) sample S2 (graph created by the authors based on experimental data).

The analysis of the experimental results shows a statistically significant correlation between R_ct_ and R_et_ values (Figure 4). For sample S1, the Pearson correlation coefficient is r = 0.821, and the coefficient of determination is R^2^ = 0.67. The regression analysis for sample S2 also indicates a positive relationship between R_ct_ and R_et_, with r = 0.72 and R^2^ = 0.52. This suggests that as thermal resistance increases, water vapor resistance also tends to increase.

Based on the results of the two-sample *t*-test for R_ct_ parameters, there is a statistically significant difference between samples S1 and S2 (t = −12.67, *p* < 0.0001). The two-sample *t*-test for Ret parameters also shows a statistically significant difference between groups S1 and S2 (t = −5.56, *p* < 0.0001). The mean value of S2 is significantly higher than S1.

In comparison with the results of thermal insulation of natural skin conducted by S.M. Çolak et al. [35], a significant difference can be observed in the thermophysiological characteristics of the analyzed materials. Namely, in their study, natural leather treated with different tanning agents (vegetable, chrome, phosphonium, etc.) had a thickness ranging from 1315 to 1388 mm, while the values of thermal resistance (R_ct_) ranged between 0.258 and 0.275 m^2^·K/W [35]. In contrast, in this study, pineapple bio-based material samples show significantly lower R_ct_ values. Overall, the bio-based material samples analyzed in this paper show significantly lower thermal insulation compared to natural leather, which can be an advantage for wear in warmer or indoor conditions.

Subjective ratings of thermal comfort were collected in three phases of testing: sitting, walking and sitting after walking. The results of metabolism and thermal flux are shown in Table 4.

As listed in Table 4, greater variability in basal metabolic rate among the subjects indicates their differences in energy expenditure. Results for working metabolism and heat flow indicate a moderate degree of deviation.

Table 5 shows the results of the subjects’ subjective assessment of thermal comfort. Subjects completed the questionnaire after each phase (after acclimatization and rest (B), after movement (M) and after sitting again and resting (E).

It was observed that comfort ratings were higher for material S2 in all three phases, with the difference being most pronounced during physical activity (walking). During testing with material S1, subjects reported a neutral sensation, while material S2 caused a subjective feeling of heat and overheating, which is consistent with its high Ret values.

The results of the subjective evaluation showed that sample S1 was rated by tested subjects as more comfortable compared to sample S2 in all phases of the test (during rest, activity and after movement). Subjects perceived sample S1 as less warm, more comfortable to wear and less stuffy, which they reflected in lower percentages of expressed discomfort and more favorable ratings.

**Figure 5:**
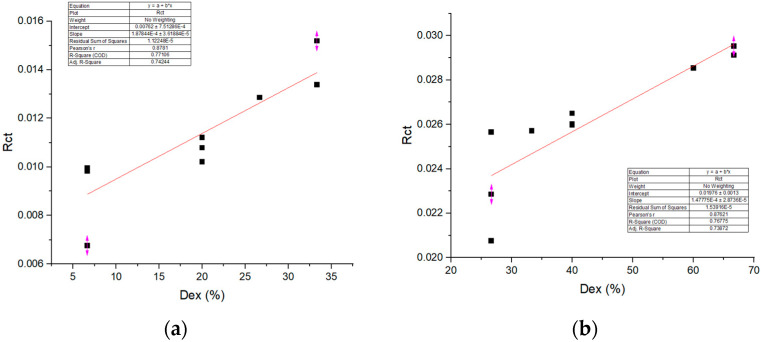
Dependence of Dex on R_ct_ for (**a**) sample S1, (**b**) sample S2 (graph created by the authors based on experimental data).

To enable correlation analysis between thermal resistance (R_ct_) and the percentage of expressed discomfort (Dex), the original 15 R_ct_ measurements were grouped and averaged into ten representative mean values. This data reduction was performed in order to match the number of Dex values and ensure a one-to-one correspondence between the two datasets. The analysis, shown in Figure 5, indicates a strong positive relationship between thermal resistance (R_ct_) and the percentage of expressed discomfort (Dex), with Pearson’s r = 0.88 and R^2^ = 0.77 for sample S1 and with Pearson’s r = 0.87 and R^2^ = 0.77 for sample S2. This suggests that higher thermal resistance is associated with increased thermal discomfort, which may be due to reduced heat dissipation from the body.

**Figure 6:**
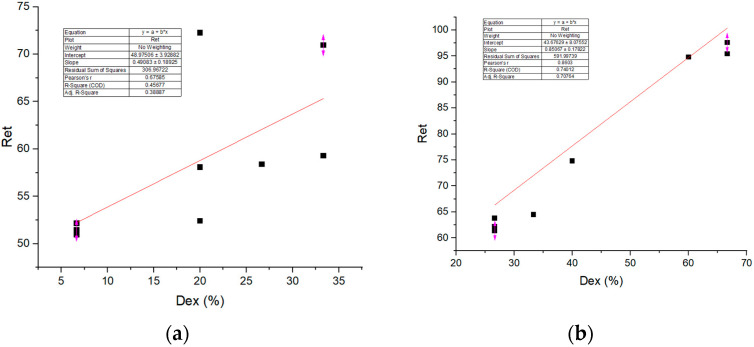
Dependence of Dex on Ret for (**a**) sample S1, (**b**) sample S2 (graph created by the authors based on experimental data).

The correlation between water vapor resistance (Ret) and the percentage of expressed discomfort (Dex), that is shown in Figure 6, indicates a strong positive relationship between thermal resistance (Ret) and the percentage of expressed discomfort (Dex), with Pearson’s r = 0,68 and R^2^ = 0.46 for sample S1 and with Pearson’s r = 0.86 and R^2^ = 0.74 for sample S2. This suggests that higher water vapor resistance is associated with increased thermal discomfort.

Although a high heat flux was measured (average 107.8 W/m^2^), subjects reported a feeling of excessive heat and discomfort. These findings indicate that heat transfer intensity alone is not sufficient to ensure thermophysiological comfort. The crucial is the material’s ability to remove water vapor and to provide properly air permeability. In situations where the resistance to the transfer of water vapor (Ret) is high, there is a retention of sweat and moisture, which creates a feeling of congestion and discomfort, despite the measured heat flux.

These subjective ratings are consistent with objective parameters. Sample S1 has a slightly lower resistance to the transfer of water vapor (Ret) but significantly better air permeability, which indicates a more efficient removal of sweat and moisture from the body surface. Although the heat flux values were relatively high, sample S1 enabled better thermal balance, because in addition to heat transfer, it also enabled better evaporation of moisture.

In contrast, sample S2 has a slightly higher water vapor resistance (Ret) and lower air permeability, which, despite physical heat transfer, caused a feeling of stuffiness and discomfort. These findings confirm that subjective comfort is not exclusively related to a single physical parameter but is the result of the interaction of several factors, including thermal insulation, vapor diffusion, ventilation and individual physiological differences of subjects.

The results of the analysis of subjective discomfort show that the sample S1 caused significantly less pronounced discomfort compared to the sample S2. The results of the two-sided oriented *t*-test indicate that there is a statistically significant difference between discomfort in the samples S1 and S2 according to the observed variable. Sample S1 has a significantly lower mean value (Dex = 17.99%, SD = 10.91) compared to sample S2 (Dex = 42.67%, SD = 16.08), t= −4.01, *p* = 0.019.

## 5. Conclusions

The research results indicate a complex connection between the subjective perception of comfort and objective physiological and thermal parameters.

The research results showed that sample S1 was more favorably evaluated in terms of thermophysiological comfort compared to sample S2, based on both subjective and objective indicators. The subjective evaluations of the subjects showed that sample S1 caused lower discomfort, less feeling of heat and a more pleasant feeling during wearing. At the same time, objective measurements confirmed that sample S1 has a slightly lower resistance to the transfer of water vapor (Ret) and higher air permeability, which enables better thermoregulation and more efficient removal of sweat and heat.

Based on the obtained results, it can be concluded that clothing items made of bio-based material can be used to make fashion pieces intended for indoor wear, but with limited application, primarily for shorter time intervals and special occasions. Long-term wearing of this material, according to the results of subjective and objective assessment of comfort, is not the most suitable due to a more pronounced feeling of thermal discomfort. The possibility of incorporating breathable fabrics in high-sweat zones can be considered from a design perspective to improve comfort while preserving the aesthetics and sustainability of the material.

Given the specific thermophysiological characteristics of the material, it is recommended that further research be directed at testing the application of pineapple skin in garments intended for outdoor, colder climates, where its ability to retain heat could be an advantage. Also, research should be extended to different designer cuts and layers in order to further evaluate the applicability of this sustainable material in contemporary fashion.

## Figures and Tables

**Table 1:** Description of analyzed materials and their basic properties.

	Pinatex Original (S1)	Pinatex Metalic (S2)
Composition	72% PALF, 18% PLA, 5% bio-PU, 5% PU	72% PALF, 18% PLA, 10% PU
Surface mass, ISO 3801:1977 [26]	392 g/m^2^	454 g/m^2^
Thickness, ISO 5084:1996 [27]	1.6 mm	1.2 mm

**Table 2:** Anthropometric data and calculated values of body surface area and ideal body mass of subjects who evaluated the skirt.

No.	$\mathrm{Body}\;\mathrm{Height}\;\left[cm\right]$	$\mathrm{Body}\;\mathrm{Weight}\;\left[kg\right]$	Age $\left[\mathrm{Year}\right]$	$\mathrm{Body}\;\mathrm{Surface}\;\mathrm{Area}\;A_{DU}\left[m^{2}\right]$	$\mathrm{Ideal}\;\mathrm{Body}\;\mathrm{Weight}\;\mathrm{IMT}\;\left[kg\right]$
1.	172	58	25	1.69	64.45
2.	167	52	24	1.57	61.20
3.	164	58	23	1.62	59.40
4.	163	60	26	1.64	59.30
5.	159	51	21	1.51	55.75
6.	170	58	24	1.67	63.00
7.	167	64	26	1.72	61.70
8.	169	63	24	1.72	62.40
9.	171	54	21	1.61	62.85
10.	165	55	20	1.59	59.00

**Table 3:** Termophisiological properties of Pinatex textile materials.

Tested Parameters	Symbol	Units	Pinatex Original (S1)	SD (S1) (%)	Pinatex Metalic (S2)	SD (S2) (%)
Air permeability, ISO 9237	AR	[mm/s]	11.3	28	2.65	28.73
Thermal conductivity coefficient	λ	[W/mK]	0.016	13.57	0.012	14.42
Thermal resistance—ISO 11092	R_ct_	$\left[m^{2}K/W\right]$	0.0106	17.54	0.0257	16.06
Water vapor resistance—ISO 11092	R_et_	[Pa^2^/W]	60.57	14.24	84.80	16.97

**Table 4:** Metabolic values of the tested subjects under real-life conditions.

No.	Basal Metabolism [kJ/min]	Working Metabolism [kJ/min]	Total Metabolism	Heat Flux [W/m^2^]
1.	3.184	7.691	10.875	107.269
2.	2.994	7.243	10.237	108.695
3.	3.168	7.613	10.781	110.938
4.	3.236	7.798	11.034	112.157
5.	2.841	7.178	10.019	110.607
6.	3.263	7.683	10.946	109.263
7.	2.456	6.876	9.332	90.444
8.	2.466	6.886	9.352	90.638
9.	2.183	6.603	8.786	90.971
10.	3.672	6.949	10.621	99.467
SD [%]	14.73	5.52	7.42	8.47

**Table 5:** Results of subjective assessment of thermal comfort regarding the questionnaire.

	Questions:	A			B			C			D			E			D_ex_
	Assessment Phase	B	M	E	B	M	E	B	M	E	B	M	E	B	M	E	Discomfort [%]
S1	Person 1	0	1	0	0	1	0	0	0	−1	0	0	0	0	0	0	20
	Person 2	0	0	0	0	0	0	0	0	0	0	0	0	0	0	0	6.66
	Person 3	0	1	0	0	1	0	0	0	−1	0	0	0	0	0	0	20
	Person 4	0	1	0	0	1	0	0	−1	0	0	0	0	0	1	0	26.66
	Person 5	0	0	0	0	0	0	0	0	−1	0	0	0	0	0	0	6.66
	Person 6	0	1	0	0	1	0	0	−1	−1	0	0	0	0	1	0	33.33
	Person 7	0	1	0	0	1	0	0	0	−1	0	0	0	0	0	0	20
	Person 8	0	0	0	0	1	0	0	0	0	0	0	0	0	0	0	6.66
	Person 9	0	1	0	0	0	0	0	0	0	0	0	0	0	0	0	6.66
	Person 10	0	1	1	0	1	0	0	−1	0	0	0	0	0	1	0	33.33
D_ex_ = 17.99%																	
S2	Person 1	0	1	1	0	1	0	0	0	0	0	0	0	0	1	0	26.66
	Person 2	0	2	1	0	2	0	0	−1	−1	0	0	0	0	0	0	33.33
	Person 3	1	1	1	1	1	0	0	1	0	0	0	0	0	1	0	40.00
	Person 4	0	2	2	1	2	1	0	−2	−2	0	1	0	0	1	1	66.67
	Person 5	0	1	1	1	1	0	0	1	0	0	0	0	0	0	0	26.66
	Person 6	0	2	2	1	2	1	0	−2	−2	0	1	0	0	1	1	66.67
	Person 7	0	1	1	0	1	0	0	1	1	0	0	0	0	1	0	40.00
	Person 8	0	2	1	0	2	1	0	1	0	0	0	0	0	1	0	40.00
	Person 9	0	1	1	0	1	0	0	1	0	0	0	0	0	0	0	26.66
	Person 10	0	2	2	0	2	1	0	−2	−2	0	1	0	0	1	1	60.00
D_ex_ = 42.67%																	

Legend: B—beginning of the test, M—middle of the test, E—end of the test, D_ex_—discomfort. A, B, C, D, E are the questions from the questionnaire.

## Data Availability

The original contributions presented in the study are included in the article. Further inquiries can be directed to the corresponding author.
